# Unified Compact ECC-AES Co-Processor with Group-Key Support for IoT Devices in Wireless Sensor Networks

**DOI:** 10.3390/s18010251

**Published:** 2018-01-16

**Authors:** Luis Parrilla, Encarnación Castillo, Juan A. López-Ramos, José A. Álvarez-Bermejo, Antonio García, Diego P. Morales

**Affiliations:** 1 Dpto. Electrónica y Tecnología de Computadores, Universidad de Granada, 18071 Granada, Spain; encas@ditec.ugr.es (E.C.); grios@ugr.es (A.G.); diegopm@ugr.es (D.P.M.); 2 Dpto. Matemáticas, Universidad de Almería, 04120 Almería , Spain; jlopez@ual.es; 3 Dpto. Informática, Universidad de Almería, 04120 Almería, Spain; jaberme@ual.es

**Keywords:** elliptic curve cryptography, AES, IoT, WSN, cryptographic processor, FPGA

## Abstract

Security is a critical challenge for the effective expansion of all new emerging applications in the Internet of Things paradigm. Therefore, it is necessary to define and implement different mechanisms for guaranteeing security and privacy of data interchanged within the multiple wireless sensor networks being part of the Internet of Things. However, in this context, low power and low area are required, limiting the resources available for security and thus hindering the implementation of adequate security protocols. Group keys can save resources and communications bandwidth, but should be combined with public key cryptography to be really secure. In this paper, a compact and unified co-processor for enabling Elliptic Curve Cryptography along to Advanced Encryption Standard with low area requirements and Group-Key support is presented. The designed co-processor allows securing wireless sensor networks with independence of the communications protocols used. With an area occupancy of only 2101 LUTs over Spartan 6 devices from Xilinx, it requires 15% less area while achieving near 490% better performance when compared to cryptoprocessors with similar features in the literature.

## 1. Introduction

The rapid evolution of Internet of Things (IoT) will lead in the coming years to important changes in everyday life for people. In fact, new IoT applications are appearing daily, taking advantage of connectivity of smart devices, thus providing new features and services for industry, finance, or the final user. Nevertheless, the enthusiasm about these new features is making engineers and companies not fully address the threats and risks to security and privacy that this wide connectivity of things poses. Every data collected by an IoT device (temperature, humidity, power consumption, etc.) can be useful for criminals for obtaining information about people being at home, or work. Therefore, in a globally interconnected world where cybercrime growths every year, security in IoT will be a critical challenge for its success and effective deployment [1,2,3,4,5]. In [1], security and privacy requirements for IoT along with legal considerations are revised. In [2,3], features of IoT are described, and security issues of distributed systems are analyzed. In [4], Physically Unclonable Functions (PUF), are proposed as an alternative for providing security in IoT devices. In [5], a survey of security protocols with application to IoT is presented.

**Figure 1:**
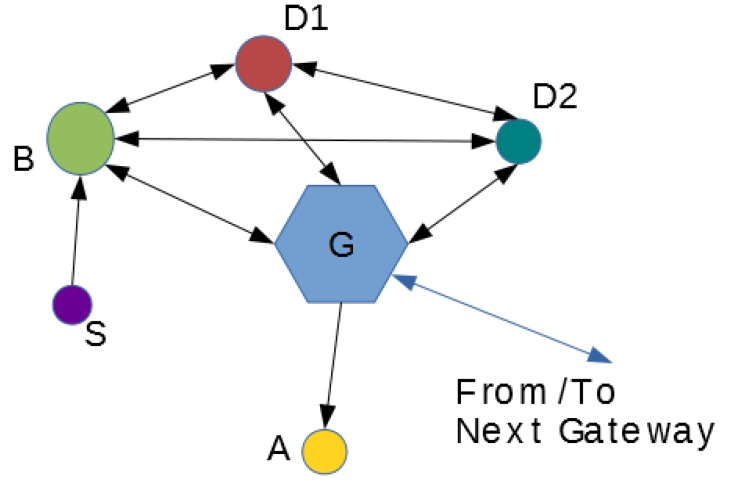
Local “Wireless Sensor Network” in the IoT environment.

Probably, the most under-protected environments in IoT are local networks consisting of low-cost smart devices used in domotics, offices, cars, or industrial automation. For these devices, the target is to provide great features and connectivity while maintaining an affordable prize. Security has been until now only a secondary objective for these devices because no personal data are interchanged, but, in the information era, all information transmitted by final-user devices is important. In fact, information generated by wireless motion sensors for lighting at home could be easily captured by a criminal, thus obtaining information about if we are or not at home. Therefore, all information interchanged by IoT devices should be protected, no matter if it is being sent through Internet gateways or among internal devices in a Local Wireless Network. Although devices used in domotics or automotive are not only sensors, but actuators too, the local networks formed by them can be treated as Wireless Sensor Networks (WSN), and we use the term “Local Wireless Sensor Networks” in the following. Figure 1 shows a typical WSN into the IoT context, with different types of devices interconnected:

- *S* is a Sensor device only generating information that can be collected and processed by smart device *B*.
- *B* is a smart device that can generate and receive information from different devices (*S*, *D1*, *D2*), and communicate with the Gateway *G* for accessing the Internet.
- *D1* and *D2* are smart devices with sensors and programmable features that can be controlled by *B* or devices from the Internet through Gateway *G*.
- *A* is an actuator device that can be remotely controlled from the Internet through Gateway *G*.
- *G* is a Gateway providing access to the elements in the WSN to the Internet.

Therefore, in the IoT, we have millions of Local WSNs interconnected, and within each WSN there are different types of devices interchanging information using different network protocols (Wifi, Bluetooth, Zigbee, etc.). Each of these protocols provides different levels of security, thus making difficult to establish a minimum security level in such a heterogeneous set of devices, protocols and networks. In this context, a security procedure independent of the network protocol is desirable.

In fact, there are several works on security in WSNs [6,7,8,9], proposing to secure communications among the different interconnected devices while not harming performance and/or features. In [6,7], the need of a secure and efficient key-distribution mechanism is stated, while, in [8], a secure and DoS-Resistant broadcast authentication protocol is proposed. In [9], the use of Identity Based Cryptography for avoiding a Public-Key infrastructure is proposed. The majority of these procotols are based on the encryption of communications using Advanced Encryption Standard (AES) [10] or any other symmetric cryptosystem. Symmetric cryptography does not require high computing resources, therefore it can be included into low-cost and low-power devices without excessive drawbacks. Nevertheless, the interchange of these symmetric keys through an insecure environment such as wireless communications over unguided mediums should be solved by means of public-key cryptography. However, public-key cryptosystems such as Rivest–Shamir–Adleman (RSA) [11,12] or Elliptic Curve Cryptography (ECC) [13,14] require high computing resources, and several alternative solutions [15,16,17] have been proposed in order to avoid the computation overhead associated to the use of public-key cryptography. These solutions are based on pre-distribution of keys, with different degrees of sophistication for increasing security. In any case, Public-key Cryptography is the most secure solution for key exchange in wireless communications, although it may involve high computing resources. In addition, if there is a large number of devices connected, high memory resources can be necessary for storing all the keys. In this work, we propose the use of a Group Key protocol [18] for overcoming these inconveniences, enabling the use of ECC for WSNs interconnecting IoT Devices. Moreover, we present the design of a very compact ECC-AES cryptographic co-processor providing a complete solution for securing WSNs communications using Group Keys protocols. A proof of concept is provided implementing the co-processor along with a 8-bit microprocessor, communication interfaces, RAM memory, and I/O ports in a low-cost Spartan 6 LX9 microboard [19]. The rest of the manuscript is organized as follows: Section 2 introduces security issues in Local Wireless Sensor Networks. Section 3 details usefulness of Elliptic Curve Cryptography for IoT devices connected to Wireless Sensor Networks. Section 4 is devoted to the description of the proposed compact cryptoprocessor for IoT devices. Section 5 presents the experimental results, and Section 6 describes the conclusions of the paper.

## 2. Security in Local Wireless Sensor Networks

Security in a Local WSN depends on several aspects, as outlined in [6,7], that can be summarized in three issues: encryption of communications, authentication and hardware/software protection. The following subsections analyze them.

### 2.1. Encryption of Communications

When using wireless communications, encryption of communications is basic for ensuring security and privacy [6]. Symmetric cryptosystems such as AES (block cipher) [10] or Trivium (stream cipher) [20] can provide secure connections among the different nodes of a Local WSN without severe computation overheads. Additionally, it is necessary that the nodes involved in such a communication accord a shared secret key for interchanging the encrypted information. The optimal way for sharing secret keys from a security point of view is the use of a public-key cryptosystem such as RSA or ECC. However, implementation of public-key cryptosystems results in high computing requirements which are difficult to assume for the low-cost Microprocessor Units (MPU) used in IoT devices [15]. In this scenario, several methods for distributing keys have been proposed, which can be classified into three groups:

- Pre-shared keys [15,16]. The simplest method for distributing keys is to use a pre-shared key stored in the involved nodes before deployment. This method has the advantage of minimal memory and computation requirements, but also has three important drawbacks: if one of the nodes is compromised, the entire network will be compromised, and keys can not be changed without updating the firmware/software of all the nodes. Moreover, the use of the same key for all communications gives a lot of information to attackers in order to derive the key.
- List of pre-shared keys [15]. The method of pre-sharing a key can be improved by distributing a list of pre-shared keys to the nodes. In this case, any of the keys can be used in communications, thus making the derivation of the key by eavesdropping more difficult at the expense of more memory requirements. The other two drawbacks are not solved: key change is limited to the pre-shared list, thus if one node is compromised, again the entire network is compromised.
- Random and Multipath methods. More sophisticated proposals for distributing pre-shared keys based on random lists and multipath methods [17] provide mechanisms for avoiding the compromising of the entire network when one/some nodes is/are jeopardized. These methods are based on symmetric-key cryptosystems.

In any case, public key cryptography is the best method for distributing keys, although it implies computing times of tens of seconds for calculating a scalar-point ECC operation in software implementations used in sensor nodes such as MICA2 [21].

### 2.2. Authentication

Authentication is required in order to ensure that a node is authorized to access the WSN, and for guaranteeing that the content, origin and destiny of a message are trusted. Recently, several authentication methods have been published [8,22,23], providing key-agreement schemes and signatures based on ECC. In general, ECC is preferred in WSNs because of the shorter keys needed when compared to other solutions like RSA [24,25], thus being specially suitable when narrow bandwidths and limited memory resources are available [26]. In fact, a 163-bit ECC cryptosystem provides the same security as 1024-bit RSA, with a significant saving in communications and processing. Emerging techniques such us reputation metrics, used in Point-to-Point (P2P) networks [27,28], could also be an option for authentication and key delivery in WSNs.

### 2.3. Hardware/Software Protection

Hardware/software protection refers to protection against attacks for shutting down the hardware of the IoT device, scaling privileges for taking the control of the device, altering functionality, etc. At the software level, the operating system is the last defence for avoiding attackers taking control of the device. However, limited memory and hardware resources make difficult developing an operating system with multiple virtual memory spaces. In TinyOS [29], a link layer architecture called TinySec [30] has been included, but, in any case, a great effort should be done when programming applications in order to avoid stack overflow and similar attacks.

At hardware level, again limitations in memory and hardware resources imply the use of simple MPUs without security infrastructures. There are several hardware-protection methods that can provide hardware verification by means of a digital signature [31,32] or specific MPU-protection procedures [33], but they only detect modifications in the hardware. In [34], a method for hardware activation is proposed, blocking the hardware if it has been modified. In this case, ECC is also required.

## 3. ECC Cryptography for IoT WSNs

As shown in the previous section, Elliptic Curve Cryptography is desirable for all aspects of security in Local WSNs used in IoT. Therefore, to maintain high security levels, independently of the protocol used for wireless communications, ECC capabilities should be enabled. They can be provided by means of software libraries such as TinySec [30], or if cost and/or power consumption are important but not critical, by means of a hardware coprocessor. Hardware implementations of ECC present two main advantages:

- ECC operations are accelerated, thus allowing keys to be renewed frequently.
- MPU is freed of complex cryptographic operations.

Therefore, a hardware coprocessor for ECC allows to continue using low-cost MPUs while maintaining performance of the IoT device, and enabling high levels of security.

### 3.1. ECC Key Management

An important aspect of key sharing among IoT devices forming a WSN is how the keys are managed in the network. The simplest scheme for sharing keys in ECC is the ECC Diffie–Helman algorithm (ECDH). It is used as an example in CoAP [5] security using Datagram Transport Layer Security (DTLS), where ECC is adopted to support the RawPublicKey and Certificates security modes, enabling key agreement using the Elliptic Curve Diffie–Hellman Algorithm with Ephemeral keys (ECDHE). Nevertheless, ECDH and ECDHE are used for sharing keys between two nodes, being required to store *n* public keys for ensuring communications in a Local WSN with *n* nodes. A better option for sharing keys in a Local WSN is the use of a Group Key Management protocol, where the same key is shared by all of the members of the Local WSN. The next subsection presents a group key protocol specially designed for Local WSNs.

### 3.2. Group Key Protocol for Local WSNs

The following describes a Group Key Management protocol over elliptic curves. The protocol is distributed, i.e., there is no central authority that rekeys the group and the shared key is built using every user’s private key. The general case over an arbitrary group was introduced in [18]. Its security is based on the Decisional Diffie–Hellman Problem in the group.

Users $U_{j}$, j=1,…,n agree on an elliptic curve and a generator *P* of *E*. We are assuming that the user who acts as a key manager node in the set up stage is user $U_{n}$. Then, every user $U_{j}$, j=1,…,n−1 makes public a pair of points $\left(r_{j}P,x_{j}P\right)$. The pair of integers $\left(r_{j},x_{j}\right)$ constitutes the $U_{j}$’s private key. Then, the following steps are completed:

- User $U_{n}$ computes the first common key $K_{1}=r_{n}(\sum_{j=1}^{n-1}r_{j}P)$.
- User $U_{n}$ computes ${\left\{r_{n}(\sum_{j=1,j\neq i}^{n-1}r_{j}P)\right\}}_{i=1}^{n-1}$ and chooses $\left(r_{n}^{\prime },x_{n}^{\prime }\right)$ that will be his new private key.
- User $U_{n}$ broadcasts $\left\{Y_{1,1},\ldots ,Y_{1,n-1},Y_{1,n},R_{1},S_{1}\right\}=\{r_{n}(\sum_{j=2}^{n-1}r_{j}P)-x_{n}x_{1}P,$ $r_{n}(\sum_{j=1,j\neq 2}^{n-1}r_{j}P)-x_{n}x_{2}P,\ldots ,r_{n}(\sum_{j=1}^{n-2}r_{j}P)-x_{n}x_{n-1}P,K_{1}-r_{n}^{\prime }r_{n}P-x_{n}^{\prime }x_{n}P,r_{n}P,x_{n}P\}$
- Every user $U_{j}$, j=1,…,n−1 computes $Y_{1,j}+r_{j}R_{1}+x_{j}S_{1}=K_{1}$

Let us assume now that user $U_{i}$ sends a rekeying message. This is made in the following way assuming that the user stores the keying message:

$$\left\{Y_{1,1},\ldots ,Y_{1,n-1},Y_{1,n},R_{1},S_{1}\right\}$$

- User $U_{i}$ chooses a new private key given by $\left(r_{i}^{\prime },x_{i}^{\prime }\right)$.
- User $U_{i}$ computes the new key $K_{2}=r_{i}^{\prime }K_{1}=r_{i}^{\prime }r_{n}(\sum_{j=1}^{n-1}r_{j}P)$.
- User $U_{i}$ broadcasts the rekeying message
  $$\left\{Y_{2,1},Y_{2,2},\ldots ,Y_{2,i},\ldots ,Y_{2,n},R_{2},S_{2}\right\}=\left\{r_{i}^{\prime }Y_{1,1},r_{i}^{\prime }Y_{1,2},\ldots ,K_{1}-r_{i}^{\prime }r_{i}^{\prime }R_{1}-r_{i}^{\prime }x_{i}^{\prime }S_{1},\ldots ,r_{i}^{\prime }Y_{1,n},r_{i}^{\prime }R_{1},r_{i}^{\prime }S_{1}\right\}$$
- Every user $U_{j}$, j=1,…,n,j≠i computes $Y_{2,j}+r_{j}R_{2}+x_{j}S_{2}=K_{2}$.

The next rekeying messages are created in the same way by any member of the communication group.

It should be noted that the operations required by this protocol are scalar-point multiplication and point addition/subtraction. This last operation is not usually available in ECC cryptoprocessors.

## 4. ECC163AES128 Cryptoprocessor for IoT Devices

As has been pointed out in previous sections, ECC is the best option for key distribution in Local WSNs of IoT devices. Its high-computation requirements can be overcome with a hardware-implementation of the ECC cryptosystem, thus avoiding IoT devices MPUs being overhead. In this sense, ECC cryptosystems can be defined over binary fields [35,36,37], enabling efficient hardware implementations [38,39,40,41,42].

On the other hand, FGPAs are emerging as a useful solution for implementing sensor nodes when power consumption/cost are not critical [43]. These programmable devices present important advantages related to their reconfiguration capabilities, thus allowing easily updating cryptographic algorithms if they are broken [44]. Therefore, security vulnerabilities can be solved by means of a firmware update of the IoT devices, instead of redesigning or substituting physical devices. Moreover, the research advances on such devices are leading to a continuous improvement in power consumption and moderation in costs [43]. Taking into account these considerations, in this article we present a very compact cryptoprocessor for securing IoT devices operating in a Local WSNs. The cryptoprocessor has been designed for providing a complete cryptographic framework, thus incorporating a symmetric cryptosystem (AES-128), together with a public-key ECC cryptosystem with group-key support. This cryptographic processor, named ECC163AES128, unlike the others in the literature, shares resources between the AES-128 and the ECC-163 implementations, obtaining an unified architecture that achieves minimal resources occupation in FPGA devices. The target device for our design has been a low-cost Spartan 6 LX-9 device [19] with only 5720 LUts from Xilinx, demonstrating that a complete design including an 8-bit MPU, UART peripherals and ECC163AES128 can co-exist in the same chip. It should be noted that ECC163AES128 is intended to be used in IoT devices installed at home or office. In this context, it has been assumed that attackers has no physical access to the devices, thus orienting the design to saving area resources rather than protecting the cryptoprocessor against side-channel attacks.

The main features of ECC163AES128 can be summarized as:

- *ECC support for curves over $GF(2^{163})$ field included in the FIPS standard [37], for achieving high security levels.* To achieve lower area resources, we have selected the minor-size field available in ECC standards [35,36,37], but providing guaranteed security levels. We have also included support for pseudo-random curves, and not only Koblitz as it is usual in ECC co-processors.
- *Group key management support.* This implies to make available point addition/subtraction additionally to scalar-point operation.
- *AES support.* For freeing completely the MPU of cryptographic operations, we include in the same design AES-128 symmetric encryption according to standard [10].
- A 32-bit interface, for easing interconnectivity with 8-bit, 16-bit and 32-bit MPUs/CPUs

ECC163AES128 can operate in two different modes:

1. Mode 0 (ECC): The processor operates in “ECC” mode, allowing to implement an ECC public-key cryptosystem.
2. Mode 1 (AES): The processor operates in “AES” mode, allowing encrypting/decrypting using AES-128.

Table 1 shows the operations available for each mode.

**Figure 2:**
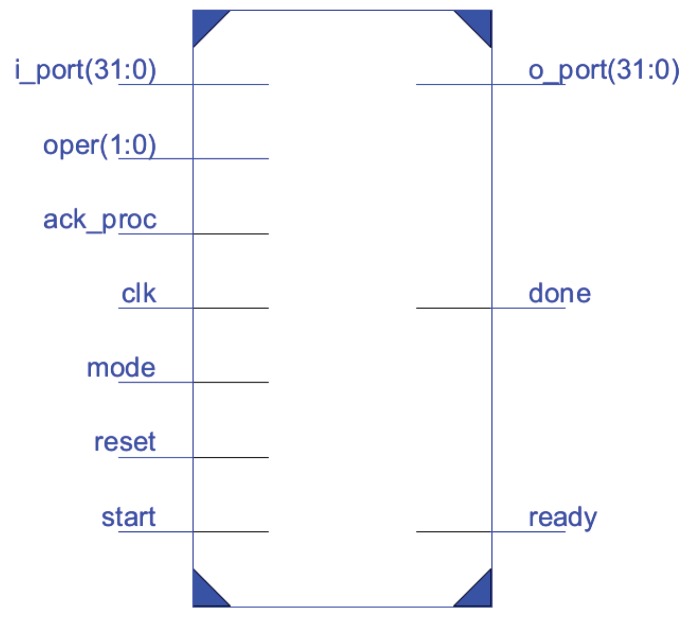
ECC163AES128 pinout.

Figure 2 shows the pinout for the ECC163AES128 core. The functions of each pin are given in Table 2.

The next subsections are devoted to describing the design and operation of the ECC163AES128 cryptographic processor.

### 4.1. AES-128 Support

**Figure 3:**
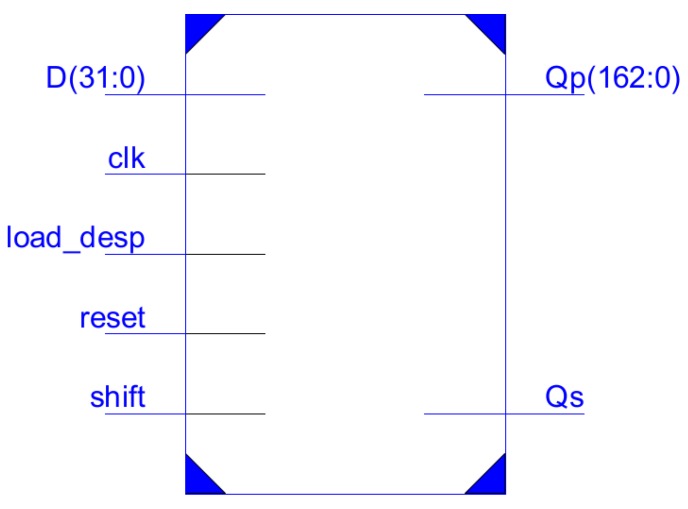
reg_interface I/O.

A compact implementation of AES-128 [10] encryption/decryption standard can be obtained following the ideas of [45], where it is proposed a 444 LUT implementation using the embedded RAM blocks available in FPGAs for saving LUTs resources. Implementation results of [45] do not include the I/O interface. To further improve area resources, we have introduced two novelties:

- First, we have designed a shared interface register between AES processing blocks, and ECC ones. This interface register has a 32-bit input, and a 163-bit output, as shown in Figure 3. In addition, it has a serial output (which will be commented later, when describing ECC-163 blocks), and control inputs for 32-bit parallel loading along to 32-bit displacement (load_desp) and 1-bit shifting (shift). The area requirement for this interface register is 163 LUTs. Note that there are no 128-bit (or 163-bit) parallel input to this register, saving 162 LUTs (if the parallel input is included, area occupancy is 325 LUTs). This area saving is 8% of the total area required by the entire cryptoprocessor.
- The absence of 128-bit parallel input in reg_interface prevents it from being used as the state register required by AES operations [10]. Therefore, the second novelty consists in introducing this register in the embedded RAM blocks, thus requiring 12 163-bit words for AES operations (11 words for key schedule and one additional for implementing the state register). Again, for optimizing resources, we will share embedded RAM blocks with ECC-163 processing blocks, it being the reason for defining a word-width of 163 bits.

**Figure 4:**
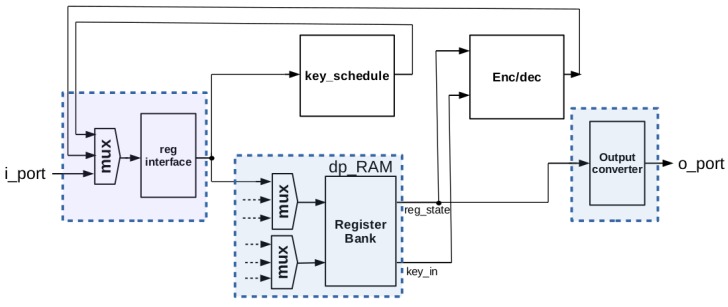
Blocks providing AES-128 support in the ECC163AES128 cryptoprocessor.

A diagram showing the blocks that provide AES-128 support is given in Figure 4, where the common interface used by the entire core, and the shared memory implementing AES-128 and ECC-163 registers have been highlighted in blue. It should be noted that the register used for interfacing the core (*reg_interface*) is used as the only intermediate register for carrying out AES operations. Key schedule is stored in *dp_RAM*, and the *reg_state* register required for AES encryption/decryption is also implemented into *dp_RAM*.

**Figure 5:**
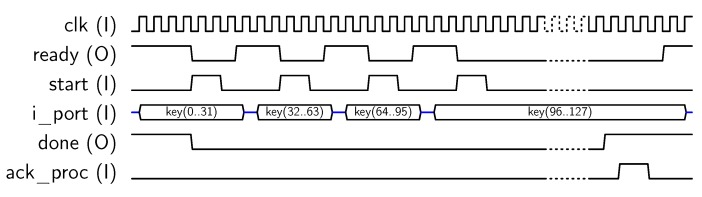
Sequence for AES-128 key input.

For using AES-128 feature, the core must be set in mode “1”, and a key schedule (operation “00”) is required prior to any encryption/decryption operation, as described in [10]. To perform *key_schedule* operations, a 128-bit private key must be provided to the core, which will calculate and store the keys to be used in each AES round into the *dp_RAM*. Later, this key schedule can be used for encrypting/decrypting 128-bit blocks. The core uses the 32-bit *key* input for introducing the key in four steps. The signal sequence for introducing the key is resumed in Figure 5, where delays introduced by the μ-processor have been included. Note that keys could be introduced only in four clock cycles, but asynchronous communication with the MPU requires at least eight clock cycles (each key block is loaded when *start* signal goes from “1” to “0”). Once the key has been loaded, the *key_schedule* operation starts. When the key schedule computation is finished, the core sets the *done* output to “1”. Then, the MPU acknowledges the end of the operation with *ack_proc* signal, as shown in Figure 5. As a result, the key schedule is stored into the internal memory, ready for use in encryption/decryption operations.

**Figure 6:**
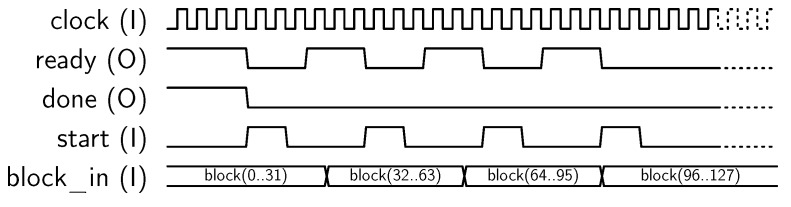
Sequence for block input.

Encryption operation is requested by setting to “01” the *oper* input. Previously, a key schedule must be generated using oper “00”, as described above. In order to encrypt a 128-bit block, it has to be loaded in four steps using the *i_port* input, as shown in Figure 6.

**Figure 7:**
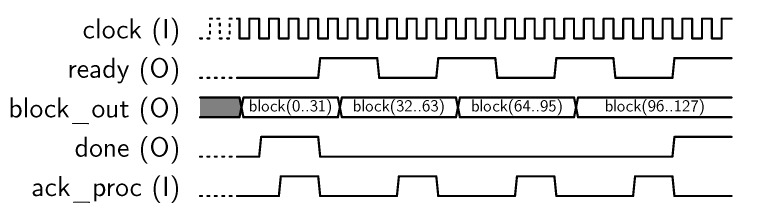
Sequence for block output.

Next, encryption operation starts, and the core sets *done* output to “1” when encryption has been completed. Finally, the encrypted 128-bit block can be recovered by reading the *o_port* 32-bit output in four steps, as shown in Figure 7.

Decryption of a 128-bit block is carried out setting *oper* to “10”, and following the same timing diagrams as for encryption operation.

### 4.2. ECC-163 Support

To provide ECC cryptosystem over binary fields ($GF(2^{m})$), the basic operation for generating a secret shared value by means of a Diffie–Helman scheme is the scalar-point operation [35].

This calculus requires three main field operations: addition, multiplication and inversion/division, being inversion the most costly [35]. In order to avoid inversion as much as possible, we have selected the Montgomery ladder algorithm over projective coordinates [46], which reduces operations to be carried out in the main loop to field additions, squarings and multiplications. If the binary representation of scalar *k* is $k=k_{m-1}2^{m-1}+,\ldots ,k_{2}2^{2}+k_{1}2+k_{0}$, and *P* is a point of a elliptic curve, Algorithm 1 provides the scalar-point product kP using Montgomery ladder algorithm [46]. This algorithm requires a fixed number of iterations to be completed, thus presenting good features against lateral attacks.

**Algorithm 1** Montgomery ladder algorithm**Require:**
*k*, *P* **Ensure:**
kP  1: $P_{1}\leftarrow P\;,\;P_{2}\leftarrow 2P$  2: **for**
i=m−2downto0
**do**  3:  **if**
$k_{i}=0$
**then**  4:   $P_{1}\leftarrow 2P_{1}\;,\;P_{2}\leftarrow P_{1}+P_{2}$  5:  **else**  6:   $P_{1}\leftarrow P_{1}+P_{2}\;,\;P_{2}\leftarrow 2P_{1}$  7:  **end if**  8: **end for**  9: **return**
$P_{1}$

Point additions of Algorithm 1 requires field inversion [35], harming performance. However, if projective coordinates are used, Algorithm 1 can be rewritten as in Algorithm 2 [46], where inversion is avoided in the main loop.

**Algorithm 2** Montgomery ladder over projective coordinates, making explicit field operations**Require:**
*k*, P(x,y) **Ensure:**
kP    1: $X_{1}\leftarrow x,Z_{1}\leftarrow 1,X_{2}\leftarrow x^{4}+b,Z_{2}\leftarrow x^{2}.\;\;Compute(P,2P)$    2: **for**
i=m−2downto0
**do**    3:  **if**
$k_{i}=0$
**then**    4:   $T\leftarrow Z_{2},Z_{2}\leftarrow {\left(X_{1}Z_{2}+X_{2}Z_{1}\right)}^{2},X_{2}\leftarrow xZ_{2}+X_{1}X_{2}Z_{1}T$    5:   $T\leftarrow X_{1},X_{1}\leftarrow X_{1}^{4}+bZ_{1}^{4},Z_{1}\leftarrow T^{2}Z_{1}^{2}$    6:  **else**    7:   $T\leftarrow Z_{1},Z_{1}\leftarrow {\left(X_{1}Z_{2}+X_{2}Z_{1}\right)}^{2},X_{1}\leftarrow xZ_{1}+X_{1}X_{2}TZ_{2}$    8:   $T\leftarrow X_{2},X_{2}\leftarrow X_{2}^{4}+bZ_{2}^{4},Z_{2}\leftarrow T^{2}Z_{2}^{2}$    9:  **end if**  10: **end for**
  11: $x_{3}\leftarrow X_{1}/Z_{1}$  12: $y_{3}\leftarrow \left(x+X_{1}/Z_{1}\right)\left[\left(X_{1}+xZ_{1}\right)\left(X_{2}+xZ_{2}\right)+\left(x^{2}+y\right)\left(Z_{1}Z_{2}\right)\right]{\left(xZ_{1}Z_{2}\right)}^{-1}+y$  13: **return**
$\left(x_{3},y_{3}\right)$

In this last algorithm, inversion/division is required only for coordinate conversion (lines 12 and 13), and the time for completing scalar-point product can be approximated by:

(1) $$T_{exec}\approx 6\times m\times T_{mul}+3\times T_{inv}+2\times T_{mul}$$

where $T_{mul}$ is the time required for a field multiplication, and $T_{inv}$ the time required for completing a field inversion/division.

Therefore, field operations required for completing Algorithm 2 are addition, squaring, multiplication, and inversion/division. In the following, implementation of each field operation is analyzed.

- *Addition*. Addition over $GF(2^{m})$ is performed by xoring bit-by-bit the binary representation of each field element. Its implementation requires *m* XOR gates.
- *Multiplication*. In order to optimize area resources, we have selected a bit-serial implementation [47] requiring only 511 LUTs in a Spartan 6 device for m=163. This implementation requires *m* clock cycles for completing multiplication (combinational multipliers such as [48,49] can perform multiplication in only one cycle but at the expense of immoderate area requirements). Digit-serial implementations can diminish the number of clock cycles, but generating an increase in area resources [41].
- *Squaring*. Squaring can be performed by means of combinational logic [50], with a slightly area increase of only 163 LUTs. Using the multiplier for squaring will result in severe performance harming. Note that in Equation (1) squaring has been considered as a combinational operation.
- *Inversion*. Inversion is the most costly operation, but usign Algorithm 2, it is required only three times. It can be computed attending to two mathematical theorems: the Extended Euclides Algorithm (EEA) and the Little Fermat Theorem (LFT). On the one hand, there are EEA implementations allowing inversion in *m* clock cycles [51,52], or digit-serial implementations [41], reducing the number of clock cycles at the expense of higher area requirements. On the other hand, the Little Fermat Theorem establishes that the multiplicative inverse in a finite field can be obtained from:
  (2) $$p^{-1}=p^{2^{m}-2}={\left(p^{2^{m-1}-1}\right)}^{2}$$
  IEEE standard 1363–2000 [35] proposes an algorithm applying successive squarings, completing the inversion in *m* clock cycles. Another possibility is the use of the Itoh–Tsujii Algorithm (ITA) [53,54], optimizing the number of steps for the exponentiation calculus. Taking into account that our design is oriented to optimize area resources, we have selected the inversion algorithm of [35], enabling the computing of inversion using multiplications and squarings, thus avoiding to introduce a specific inversion unit.

**Figure 8:**
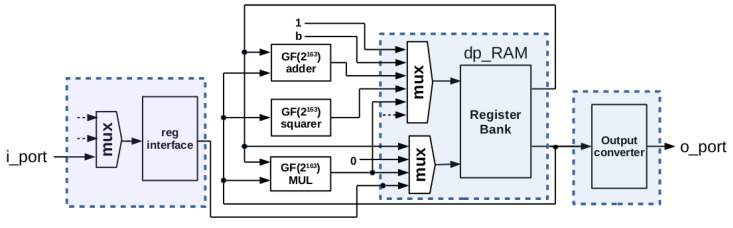
Blocks providing ECC-163 support in the ECC163AES128 cryptoprocessor.

Figure 8 shows the block diagram of the ECC-163 part of ECC163AES128 co-processor, highlighting in blue the shared elements with AES-128.

Additionally, the proposed design includes two novelties with respect to other implementations:

- The use of the shared interface register (reg_interface) as the index *k*, taking advantage of the serial output $Q_{s}$ (Figure 3) along to the control signal *shift*. This avoids the use of any other register in the processing unit.
- The register bank, implemented into embedded RAM blocks, is shared with AES support, thus making available 12 registers because of AES key schedule requirements. Therefore, there are more registers available than strictly required by Algorithm 2. Taking advantage of that, the use of pseudo-random curves can be enabled without extra area requirements.

It should be noted that implementation presented in Figure 8 includes only one multiplier, which implies that operations described in lines 4, 5, 7 and 8 of Algorithm 2 have to be executed sequentially, requiring *m* clock cycles per multiplication.

For using the ECC-163 feature, the core must be set in mode “0” (ECC). There are four different operations available in this mode: “SP_B-163” (oper “00”), “SP_Custom” (oper “01”), “PA_B-163” (oper “10”) and “PA_Custom” (oper “11”). These operations are detailed in the following sub-subsections.

#### 4.2.1. Mode “0”, Oper “00”: SP_B-163

**Figure 9:**
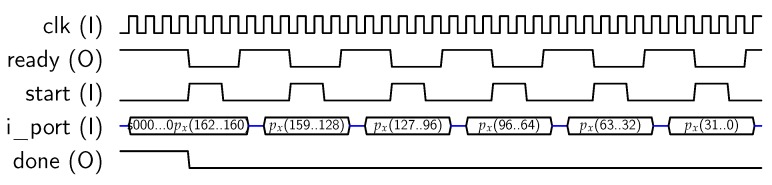
Sequence for loading $p_{x}$ input data.

**Figure 10:**
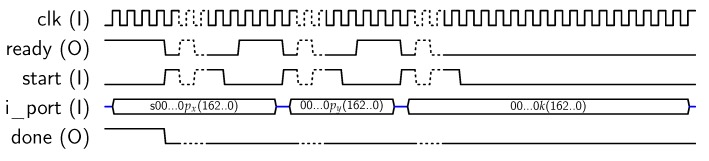
Sequence for loading $p_{x}$, $p_{y}$ and *k*.

When mode “0” is selected and “00” value is maintained in *oper* pins (see Figure 2), the core performs a Scalar-Point operation over $GF(2^{163})$, using the FIPS B163 Curve. The core expects to receive three 163-bit values, corresponding to the *x* coordinate ($p_{x}$) of the point $P=(p_{x},p_{y})$ to be multiplied, the *y* coordinate of such a point ($p_{y}$), and the scalar *k*, respectively. As the input port is 32-bit wide (*i_port* in Figure 2), each value requires six 32-bit blocks to be loaded. The first block contains the most significant bits of each value and should be padded with “0”s. The most significant bit of $p_{x}$ has an special function, because it corresponds to the sign of the scalar number. Therefore, if $p_{x}$(191) (“s” bit in Figure 9) is set to “1”, the calculus to be computed will be R=−k·P, otherwise R=k·P. Figure 9 shows the timing diagram for loading $p_{x}$. Next, $p_{y}$ and *k* must be provided to the co-processor, as shown in Figure 10.

#### 4.2.2. Mode “0”, Oper “00” Result Retrieval

**Figure 11:**
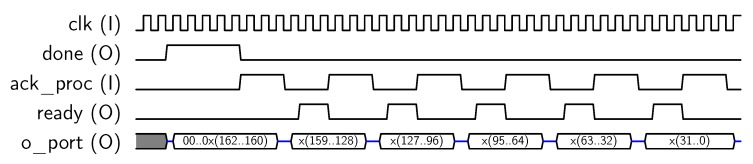
Sequence for SP ECC *x* coordinate retrieval.

**Figure 12:**
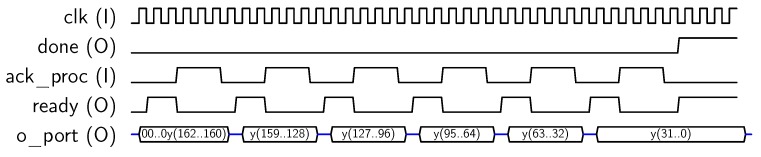
Sequence for SP ECC *y* coordinate retrieval.

After loading the last block, the cryptoprocessor starts the kP operation, signaling the calculus is completed by setting “*done*” signal to high. Then, the operation result can be retrieved from “*o_port*”. First, *x* coordinate can be retrieved as shown in Figure 11, and then *y* is available as shown in Figure 12.

#### 4.2.3. Mode “0”, Oper “01”: SP_Custom

**Figure 13:**
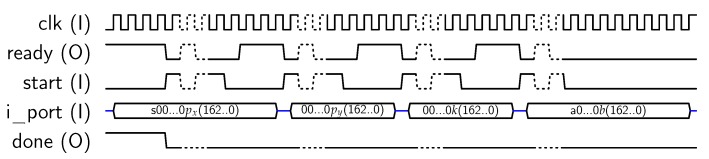
Sequence for loading $p_{x}$, $p_{y}$, *k*, *a* and *b*.

When mode “0” and oper “01” are selected (see Figure 2), the core performs an Scalar-Point operation over a custom curve in $GF(2^{163})$. In this case, the core expects to receive four 163-bit values, corresponding to the *x* coordinate ($p_{x}$) of the point $P=(p_{x},p_{y})$ to be multiplied, the *y* coordinate of such point ($p_{y}$), the scalar *k*, and the *c* parameter corresponding to the desired elliptic curve. As the input port is 32-bit wide (*i_port* in Figure 2), each value requires six 32-bits blocks to be loaded. The first block contains the most significant bits of each value and should be padded with “0”s. The most significant bit of $p_{x}$ has a special signification, because it corresponds to the sign of the scalar point. Therefore, if $p_{x}$(191) (“s” bit in Figure 10) is set to “1”, the calculus to be computed will be R=−k·P, otherwise R=k·P. In a similar way, the most significant bit of *c* corresponds to the *a* parameter of the elliptic curve, which can take the values “0” and “1”, as described in FIPS standard for the generation of pseudo-random elliptic curves, and c(162..0) corresponds to the *b* parameter of the curve. The sequence for introducing the required parameters in this mode is shown in Figure 13.

#### 4.2.4. Mode “0”, Oper “10”: PA_B-163

**Figure 14:**
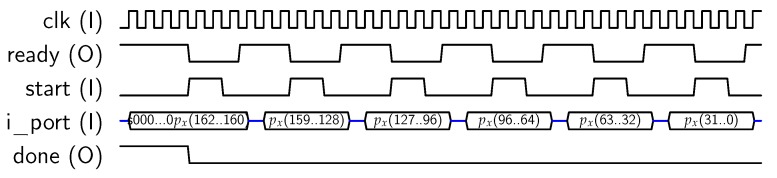
Sequence for loading $p_{x}$ input data.

**Figure 15:**
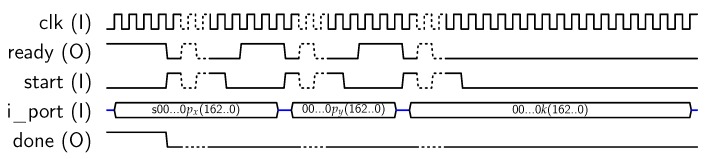
Sequence for loading $p_{x}$, $p_{y}$, $q_{x}$ and $q_{y}$.

When mode “0” is selected and “10” value is maintained in *ope*r pins (see Figure 2), the core performs a point addition over the FIPS B163 Curve. The core expects to receive four 163-bit values, corresponding to the *x* and *y* coordinates ($p_{x}$, $p_{y}$) of the first point to be added, and the *x*, *y* coordinates of the second point to be added ($q_{x}$, $q_{y}$), respectively. As the input port is 32-bit wide (“i_port” in Figure 2), each value requires six 32-bits blocks to be loaded. The first block contains the most significant bits of each value and should be padded with “0”s. The most significant bit of $p_{x}$ has a special function, because it corresponds to the sign of the point *P*. Therefore, if $p_{x}$(191) (“s” bit in Figure 14) is set to “1”, the calculus to be computed will be R=−P+Q, otherwise R=P+Q. Figure 14 shows the timing diagram for loading $p_{x}$. Next, $p_{y}$ ,$q_{x}$ and $q_{y}$ must be provided to the coprocessor, as shown in Figure 15.

### 4.3. Control Unit

The processing unit defined in the previous subsections enables AES-128 and ECC-163 support with minimal area resources. As an inconvenience, the control unit becomes very complex, requiring 272 states. Our approach for designing such a complex control unit consists in defining five sets of micro-instructions, and implementing each set in a separate ROM. The descriptions of these sets are the following:

- *IO/loading set*. This set of micro-instructions controls the loading of external data, and operations with the *reg_interfaz* register.
- *AES set*. This set includes instructions for performing AES operations.
- *ECC set*. Includes micro-instructions related to ECC operations
- *MEM_A set*. Set of instructions for exchanging values among registers in dp_RAM, using port A of dp_RAM.
- *MEM_B set*. Set of instructions for exchanging values among registers in dp_RAM, using port B of dp_RAM.

**Figure 16:**
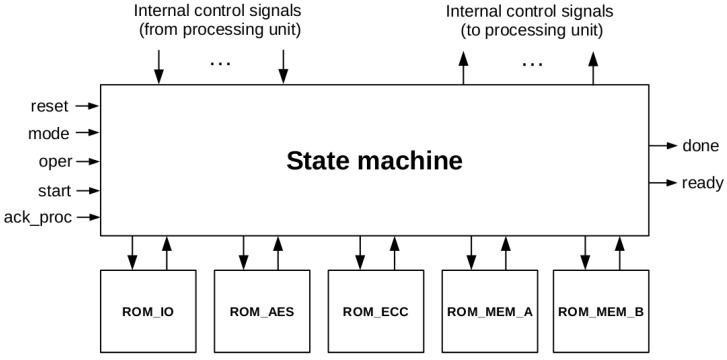
Block diagram of ECC163AES128 control unit.

Table 3 shows the first three sets of micro-instructions, with their corresponding descriptions (MEM_A and MEM_B sets are only register-transfer instructions, without special interest). Figure 16 presents the block diagram of the Control Unit.

## 5. Results

The design developed in Section 4 has been implemented in different devices from Intel (formerly Altera), and Xilinx. Table 4 presents implementation results, showing how ECC163AES128 requires only 2101 six-input LUTs in a low-cost device such as Spartan 6 xc6slx9 from Xilinx. In the case of low-cost devices from Intel (Cyclone II family), the LEs contains four-input LUTs, thus increasing the number of LEs required to 2910. On the other hand, performance is doubled when using Intel Cyclone II devices.

Table 5 shows the time required for completing the different operations provided by ECC163AES128. In this table, $t_{AES\_key}$ refers to the time required for realizing the AES key schedule at 25 MHz, 50 MHz, and at the maximum frequency supported by the design in the corresponding device. Similarly, $t_{AES\_enc/dec}$ corresponds to the time required for completing a 128-bit block AES encryption/decryption, $t_{ECC\_SP}$ corresponds to the time for completing a ECC scalar-point operation, and $t_{ECC\_PA}$ is the time for a ECC point addition. Number of clock cycles required for each operation are shown in the column heading. At a clock frequency of 50 MHz, our design can perform a 128-bit AES encryption/decryption in only 2.34 μs, and a ECC-163 scalar-point operation in 3.42 ms. Higher operating frequencies are possible, but it is not recommendable for IoT devices due to power consumption considerations. When operating at 7.38 MHz, ECC163AES128 requires 15.9 μs for AES enc/dec, and 23.2 ms for ECC scalar-point, while MICA2/MICAz [21,55] running TinyOS [29] requires 1.53 ms for AES-128 encryption (3.52 s for decryption) [55] and 34 s for ECC-163 scalar-point operation [21]. Therefore, our design operates nearly 100 times faster than software implementations for AES encryption/decryption, and nearly 1500 times when performing ECC-163 operations.

### 5.1. Comparison to Other Designs

Table 6 compares ECC163AES128 to other compact implementations of ECC and AES. For comparison purposes, clock frequency has been normalized to 10 MHz in all cases, as a typical operating frequency in sensor nodes [44].

Apart from ECC163AES128, the only design supporting ECC with scalar-point multiplication and point addition together with AES is the one presented in [44]. Relative improvements with respect to this design have been included in Table 6. In this case, our design uses 15% less LUTS, requires 50% less RAM blocks, does not use DSPs, and achieves performance improvements of 5% in AES encryption, 500% in ECC scalar-point operation, and 20% when compunting ECC point addition. The other designs are focused only on ECC, and we achieve improvements of 30% in area, while providing AES support. Designs of [39,40] provide better performance figures, but without the functionality provided by ECC163AES128, and they are prototypes without including area and performance costs of interfacing.

Therefore, the only design comparable to ECC163AES128 in area and supported operations is [44], although it should be noted that our design also provides support for random curves, and not only B-163 as in [44].

### 5.2. Proof of Concept

**Figure 17:**
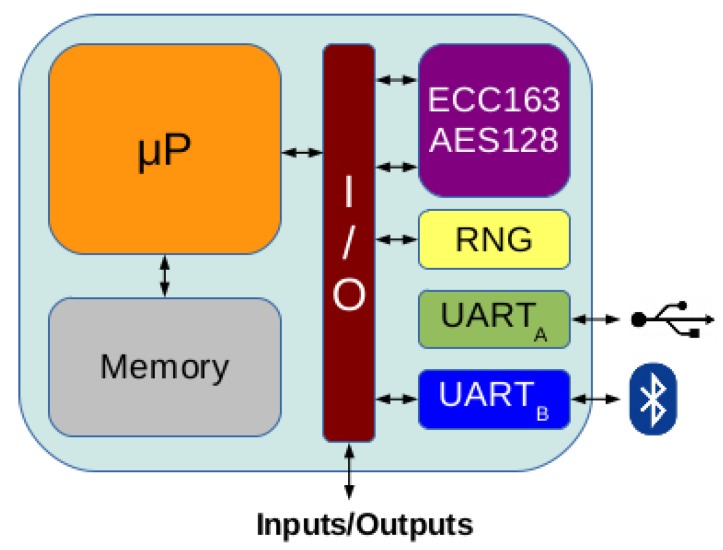
Block diagram of IoT monitoring system including ECC163AES128.

**Figure 18:**
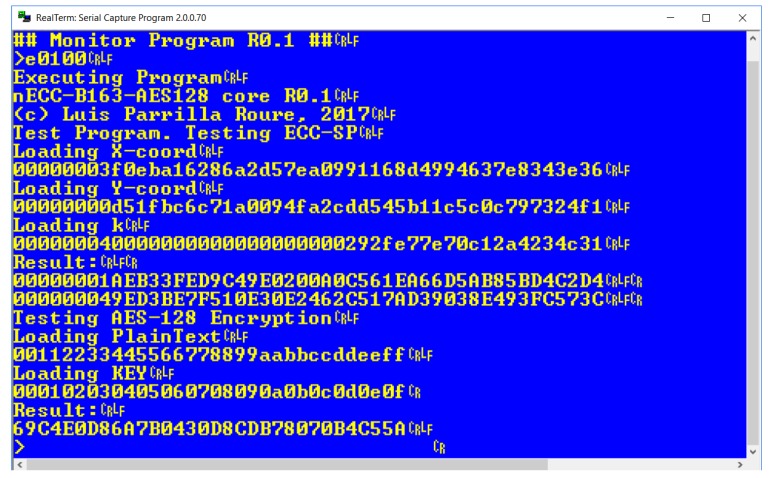
ECC163AES128 test program execution.

To prove the functionality of ECC163AES128 and its utility for being included in a low-cost IoT system, it has been implemented into a complete monitoring system connected to a Local WSN. Figure 17 shows the monitoring system, which includes a 8-bit MPU (T80 from opencores [57]), two UARTs for communications, memory, a Random Number Generator (RNG) and a I/O subsystem for attaching sensors and/or actuators. The target device is a low-cost xc6slx9-2csg324c FPGA with only 5720 LUTs from Xilinx, included into an Avnet Spartan 6 LX9 microboard [19]. Execution of a test program in the IoT platform is presented in Figure 18, showing an example of ECC scalar-point operation and other example of AES encryption.

**Figure 19:**
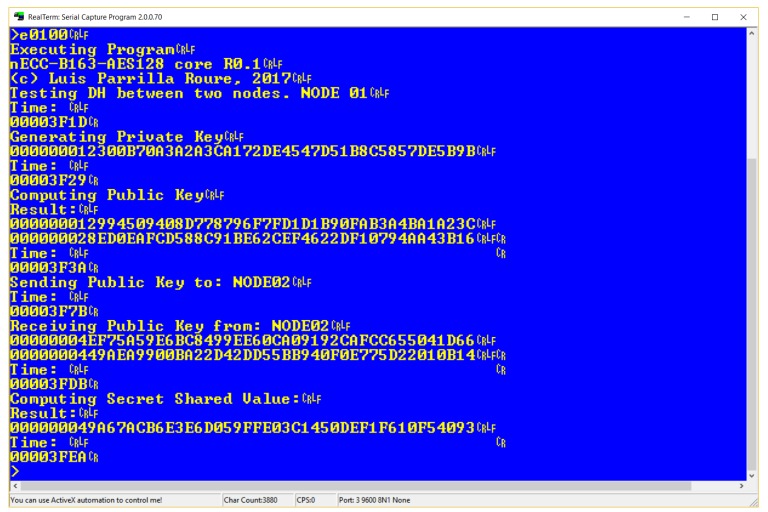
ECC163AES128 Diffie–Helmann between two nodes.

Figure 19 shows a Diffie–Hellman secret-shared value derivation [35] between two nodes using ECC163AES128 core (Node 01 output). For generating private keys, the test platform includes a RNG composed by two blocks, a True Random Number Generator (TRNG) specifically designed for FPGAs [58] feeding a Pseudo-Random Number Generator PRNG [59]. In our platform, the TRNG has been built using 50 ring oscillators [58], and the PRNG using the *rng_n1024_r32_t5_k32_s1c48* design [59], with a $2^{1024}$ period. RNG block introduces a slightly area overhead of 109 LUTs to the IoT platform. For interchanging data, a minimal network infrastructure has been developed, consisting of two Bluetooth slave modules in the sensor nodes, along with a personal computer acting as router between the two nodes. Table 7 shows time required for completing each one of the operations involved in Diffie–Hellman secret value derivation in nodes 01 and 02. Values in Table 7 include random delays introduced by network infrastructure, MPU interrupts, UART input/output, and others. As shown in this Table, time required for Diffie–Hellman protocol is around 200 ms with the platform operating at 50 MHz.

In Section 3.2, a Group Key Distribution among *n* nodes require 2n+6 scalar-point operations and n+1 point additions by the coordinator node. In addition, a temporal storing of 2n+4 points is required. If m=163, the memory requirements for n=16 nodes is around 12 KB, and 7.6 s is a good estimation of time required for completing key distribution. For n=32, around 24 KB of memory are required, and 15.2 s are needed for completing key distribution. Similar memory and time are required for rekeying. Therefore, a reasonable limit for the number or nodes to be managed using a Group Key protocol would be n=32, to maintain a contained memory usage.

## 6. Conclusions

In this article, a very compact cryptographic coprocessor that can be included into FPGA-based IoT devices has been presented. The cryptoprocessor, named ECC163AES128, provides support for symmetric ciphering using AES-128, and public-key cryptography by means of Elliptic Curve Cryptography over the $GF(2^{163})$ binary field. Moreover, ECC163AES128 provides acceleration of ECC scalar-point along with point addition, thus enabling key management by means of group keys in WSNs. Therefore, the developed cryptoprocessor allows securing heterogeneous local Wireless Sensor Networks composed of IoT devices, independently of the protocols used in wireless communications. Moreover, ECC163AES128 enables the use of Group Key Management, thus saving memory and computing resources while improving security and performance for key distribution and renewal when compared to other solutions. Finally, results show that our design requires 20% less area, while achieving 490% better performance when compared to cryptoprocessors with similar features in the literature.

## Figures and Tables

**Table 1:** ECC163AES128 modes and operations.

Mode	Oper	Operation Name	Function
	00	SP_B163	Scalar-Point operation over theNIST B-163 Curve
0	01	SP_Custom	Scalar-Point operation over a custom curve in $GF(2^{163})$
(ECC)	10	PA_B163	Point addition over the B-163 curve
	11	PA_Custom	Point addition over a custom curve in $GF(2^{163})$
	00	key_schedule	Generates the key schedule, and stores it in the RAM
1	01	encrypt	Encrypts a 128-bit block using the key schedule in memory
(AES)	10	decrypt	Decrypts a 128-bit block using the key schedule in memory
	11	reserved	Reserved for future use

**Table 2:** ECC163AES128 pin functions.

Signal	I/O	Width	Function
reset	input	1	resets the core
clk	input	1	clock input
start	input	1	control signal, starting operations
ack_proc	input	1	control signal, acknowledging data reception from the MPU
mode	input	1	selects mode of the cryptoprocessor (‘0’ for ECC, ‘1’ for AES)
oper	input	2	selects the operation to perform. (See Table 1)
i_port	input	32	data required by the core for performing the different operations
ready	output	1	control signal, indicating the core is ready for receiving data
done	output	1	control signal, indicating the core has finished an operation
o_port	output	32	output for providing the result from the operation completed by the core

**Table 3:** ECC163AES128 micro-instructions.

Set	Micro-Instruction	Function
	NOP	No I/O operation
	IREADY	output *ready* set to ‘1’
	ILOAD	*reg_interfaz* loaded with block from *i_port*
	ILOADKEY	*reg_interfaz* loaded with internal key block
	ILOADREG	*reg_interfaz* loaded with internal register block
IO/loading set	ISHIFT	*reg_interfaz* shifted
	IDONE	output *done* set to ‘1’
	IDREADY	outputs *done* and *ready* set to ‘1’
	ISETA	set value of *a* parameter of elliptic curve to FIPS B-163 value
	ILOADA	load value of *a* parameter corresponding to a custom elliptic curve
	NOPAES	No AES operation
	XORKEY	XOR with key AES operation
	BLINITAES	AES block counter initialization
	BLCNTAES	AES block counter update
AES set	ROUND_INIT	AES round counter initialization
	ROUND_CNT	AES round counter update
	BLINITRNDCNT	AES block and round counters initialization
	XORBLCNT	XOR with key and block counter update
	XORRNDCNT	XOR with key and round counter update
	NOPARITH	NO ECC operation
	BLINITECC	ECC block counter initialization
	BLCNTECC	ECC block counter updated
	CNTLOAD	ECC loop counter initialization
ECC set	CNTCOUNT	ECC loop counter update
	MULINIT	Field multiplier initialization
	MULCOUNT	Field multiplier initialization and ECC loop counter update
	INITCNTINV	Field inversion counter initialization
	CNTINV	Field inversion counter update

**Table 4:** Area and delay figures for ECC163AES128 core implementation on different devices.

Device	# LUTs/LEs	# BRAMs	Fmax (MHz)
Cyclone II			
EP2C20F484C7	2910 (LEs)	26,532 bits	103
(Intel)			
Cyclone II			
EP2C35F672C6	2983 (LEs)	26,532 bits	97.7
(Intel)			
Spartan 3AN			
xc3s700an-4fgg484	2824 (LUT4s)	11	54.9
(Xilinx)			
Spartan 6			
xc6slx9-2csg324	2101 (LUT6s)	5 RAM16 +6 RAM8	61.0
(Xilinx)			
Spartan 6			
xc6slx45t-3cfgg484	2122 (LUT6s)	5 RAM16 +6 RAM8	67.0
(Xilinx)			
Virtex 6			
xc6vlx240t-1ff1156	2121 (LUT6s)	5 RAM36+ 6 RAM18	83.8
(Xilinx)			

**Table 5:** Time required for completing each one of the available operations in ECC163AES128 core on different devices.

Device	$t_{\mathrm{AES}\_\mathrm{key}}$ 53 Cycles	$t_{\mathrm{AES}\_\mathrm{enc}/\mathrm{dec}}$ 117 Cycles	$t_{\mathrm{ECC}\_\mathrm{SP}}$ 171070 Cycles	$t_{\mathrm{ECC}\_\mathrm{PA}}$ 2174 Cycles
Cyclone II	2.12 us @25 MHZ	4.65 us @25 MHZ	6.84 ms @25 MHZ	87 us @25 MHZ
EP2C20F484C7	1.06 us @50 MHZ	2.34 us @50 MHZ	3.42 ms @50 MHZ	43.5 us @50 MHZ
(Intel)	0.52 us @Fmax	1.14 us @Fmax	1.67 ms @Fmax	21.2 us @Fmax
Cyclone II	2.12 us @25 MHZ	4.65 us @25 MHZ	6.84 ms @25 MHZ	87 us @25 MHZ
EP2C35F672C6	1.06 us @50 MHZ	2.34 us @50 MHZ	3.42 ms @50 MHZ	43.5 us @50 MHZ
(Intel)	0.54 us @Fmax	1.20 us @Fmax	1.75 ms @Fmax	22.3 us @Fmax
Spartan 3AN	2.12 us @25 MHZ	4.65 us @25 MHZ	6.84 ms @25 MHZ	87 us @25 MHZ
xc3s700an-4fgg484	1.06 us @50 MHZ	2.34 us @50 MHZ	3.42 ms @50 MHZ	43.5 us @50 MHZ
(Xilinx)	0.96 us @Fmax	2.13 us @Fmax	3.12 ms @Fmax	39.7 us @Fmax
Spartan 6	2.12 us @25 MHZ	4.65 us @25 MHZ	6.84 ms @25 MHZ	87 us @25 MHZ
xc6slx9-2csg324	1.06 us @50 MHZ	2.34 us @50 MHZ	3.42 ms @50 MHZ	43.5 us @50 MHZ
(Xilinx)	0.87 us @Fmax	1.92 us @Fmax	2.81 ms @Fmax	35.7 us @Fmax
Spartan 6	2.12 us @25 MHZ	4.65 us @25 MHZ	6.84 ms @25 MHZ	87 us @25 MHZ
SP-605	1.06 us @50 MHZ	2.34 us @50 MHZ	3.42 ms @50 MHZ	43.5 us @50 MHZ
(Xilinx)	0.79 us @Fmax	1.75 us @Fmax	2.55 ms @Fmax	32.5 us @Fmax
Virtex 6	2.12 us @25 MHZ	4.65 us @25 MHZ	6.84 ms @25 MHZ	87 us @25 MHZ
xc6vlx240t-1ff1156	1.06 us @50 MHZ	2.34 us @50 MHZ	3.42 ms @50 MHZ	43.5 us @50 MHZ
(Xilinx)	0.63 us @Fmax	1.40 us @Fmax	2.00 ms @Fmax	26.0 us @Fmax

**Table 6:** Comparison of ECC163AES128 to other compact cryptoprocessors.

Design	# LUTs/LEs	# BRAMs	AES Support	$t_{\mathrm{AES}}$	$t_{\mathrm{ECC}\_\mathrm{SP}}$	$t_{\mathrm{ECC}\_\mathrm{PA}}$
ECC163AES128		5 BRAM16				
(Spartan 6)	2101	6 BRAM8	Yes	5.3 us	17.1 ms	218 us
xc6slx9-2csg324	(100%)			(100%)	(100%)	(100%)
De la Piedra ECC-163 [44]		2 RAM36
(Artix 7)	2412	21 RAM18	Yes	5.50 us	83.9 ms	253 us
XC7A100TL	(115%)	38 DSPs		(104%)	(490%)	(116%)
Leong ECC-155 [56]	3736	–	No	–	24.9 ms	–
(Virtex E)						
XCV1000-6						
Orlando ECC-167 [39]	3002	10	No	–	1.61 ms	–
(Virtex E)						
XCV400E-8-BG-432						
Pu ECC-167 [40]	3023	10	No	–	1.58 ms	–
(Virtex E)						
XCV400E-8-BG-432						

**Table 7:** Time required for Diffie–Hellman operations between two nodes.

Operation	Time (Node 01)	Time (Node 02)
Private key generation	12 ms	11 ms
Public key derivation	17 ms	16 ms
Public key transmission	65 ms	96 ms
Public key reception	96 ms	66 ms
Secret value derivation	15 ms	16 ms
Total time	205 ms	205 ms
